# The bacterial SOS response promotes the expression of the transposase encoded by IS*CR* mobile genetic elements

**DOI:** 10.1128/jb.00551-25

**Published:** 2026-06-23

**Authors:** Claire Lallement, Thomas Jové, Cécile Pasternak, Sandra Da Re, Marie-Cécile Ploy

**Affiliations:** 1 Univ. Limoges, RESINFIT, U1092, Limoges, France; 2 INSERM RESINFIT, U1092, Limoges, France; 3 CHU Limoges, Laboratoire de Bactériologie-Virologie-Hygiène, Limoges, France; Southern University of Science and Technology, Shenzhen, Guangdong, China

**Keywords:** antibiotic resistance, *Escherichia coli*, insertion sequence, SOS response

## Abstract

Insertion sequences (IS) are widely involved in bacterial genomic plasticity by disrupting, adding, moving genomic sequences, or by activating or extinguishing gene expression. A specific family of IS, IS*CR* (for insertion sequence of Common Region), is thought to be involved in the dissemination of antibiotic resistance genes (ARGs). While some IS*CR* members are commonly found in bacteria isolated in clinical settings and can contribute to downstream ARG expression, the mechanisms regulating the IS*CR*-encoded transposase expression have remained uncharacterized. Here, we investigated the expression of the transposase genes of IS*CR1*, IS*CR2,* and IS*CR8* and its regulation in *Escherichia coli*. Using *in silico* analyses and *in vitro* experiments, we showed that the expression levels were extremely low, as observed for most IS transposases. We further demonstrated the direct role of DNA damages and the key SOS response repressor, LexA, in controlling the activity of the transposase promoter. These results provide evidence that the mobility of at least some IS*CR* elements may be promoted upon bacterial exposure to antibiotics inducing the SOS response.

**IMPORTANCE:**

Mobile genetic elements (MGEs) are the most prevalent cause of antibiotic resistance emergence. Among these mobile elements, insertion sequences (IS) are well known to allow the dissemination of antibiotic resistance genes (ARGs) through the action of their transposase. Here, we studied the regulation of the transposase expression in a specific family of IS, the IS*CR* family, some members of which are known to be involved in antibiotic resistance. Characterizing the regulation of transposase expression is an important starting point for understanding how these IS can contribute, through their movement, to the spread and expression of antibiotic resistance.

## INTRODUCTION

Bacterial mobile genetic elements (MGEs) are key players in the dissemination of antibiotic resistance genes (ARGs) through horizontal gene transfer (1). Insertion sequences (IS) are the most widespread type of MGEs and encode all necessary material for their transposition (2). IS can differently impact the physiology of their host cells by moving, disrupting, or activating genes (3). IS elements are generally tightly regulated through diverse mechanisms acting at different levels, including transcriptional, translational, and/or post-translational levels (reviewed in reference 4). For instance, the transposition of IS*10* is controlled by the SOS response in *Escherichia coli* (5), the transposition of IS*2* by the global regulator CRP (cAMP receptor protein) (6), and the transposition of IS*50* is regulated by Hfq (mediated through CRP) and the sRNA SgrS (7).

**Fig 1:**
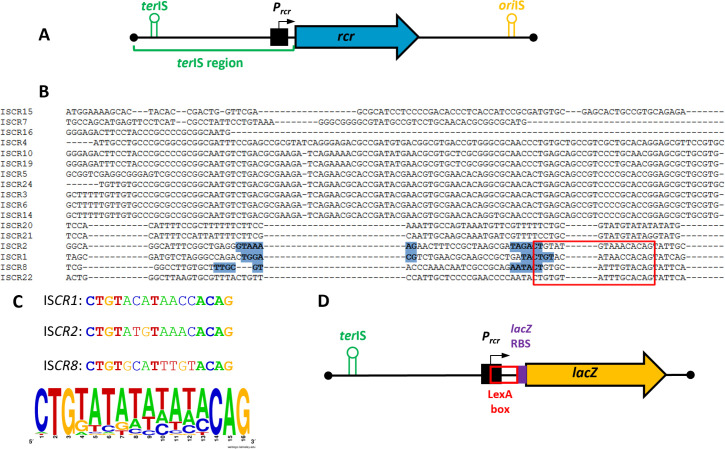
Structure of IS*CR* and alignment of their *ter*IS regions (**A**) General structure of IS*CR*. IS*CR* consists of an *rcr* transposase gene and its promoter P*_rcr_*, bound by two pairs of inverted repeats, namely, *ter*IS and *oriI*S. (**B**) Alignment of *ter*IS regions. Putative −35 and −10 σ^70^ elements of *rcr* promoters of interest are shown in bold and highlighted in blue. Putative LexA box is boxed in red. Aligned sequences include the *ter*IS region (up to the *rcr* start codon) limited to 150 bp. Alignment made with Clustal Omega (https://www.ebi.ac.uk/jdispatcher/msa/clustalo) with default parameters. (**C**) Comparison of LexA operators in IS*CR1,* IS*CR2,* and IS*CR8.* Weblogo was used to generate the LexA box consensus sequence (12) from a set of 28 experimentally verified *Escherichia coli* LexA-binding sites (listed in reference 13). Bases identical in each of the three predicted IS*CR* LexA-binding sites and matching the LexA box logo are given in bold. Each color represents a nucleotide (blue: cystosine, red: thymine, yellow: guanine, green: adenine) (**D**) Transcriptional fusion used to assess P*_rcr_* activity in this study. Reporter gene *lacZ*, encoding for β-galactosidase, was fused in a translational fusion with native (P*_rcr_*), LexA-box-mutated (P*_rcr_*L), or −10 region-mutated (P*_rcr_*M) promoters of IS*CR1*, IS*CR2,* and IS*CR8*.

IS of the Insertion Sequence Common Region (IS*CR*) group are of clinical concern since they are commonly adjacent to multiple ARGs or pathogenicity regions (8). IS*CR* elements are related to the IS*91* family members with which they share several features. These include (i) a gene encoding a transposase of the HUH superfamily of single-strand nuclease (hereafter referred to as *rcr*, according to reference 9) and (ii) two distinct ends that include sequences forming secondary structures, namely, *ori*IS and *ter*IS, where the transposition process initiates and terminates, respectively (8, 10) (Fig. 1A). These specificities suggest that IS*CR* elements may transpose by a rolling-circle mechanism as presumed for IS*91* and can therefore transpose adjacent genes (8, 11).

The prevailing IS*CR* element, namely, IS*CR1*, is exclusively found downstream of the 3′ conserved region associated with class 1 integrons and displays a large diversity of adjacent genes, especially ARGs (8, 9). Moreover, IS*CR1* harbors two outwardly oriented promoters (P_OUT_) in its *ori*IS end (14–16), contributing to the expression of downstream ARGs (9). Additionally, two other IS*CR*s commonly found in the literature and databases are (i) IS*CR2*, which is mainly described adjacent to ARG within integrative conjugative elements (ICEs), especially the SXT element (17), and (ii) IS*CR8,* which is found as multiple copies in environmental bacterial plasmids (18).

Despite their potential importance in clinical settings, nothing is known about the regulation of the expression of IS*CR* elements.

In this study, we characterized, both *in silico* and *in vitro*, the key DNA motifs involved in the regulated expression of the *rcr* transposase-encoding genes of three members of the IS*CR* group (IS*CR1*, -*CR2,* and -*CR8*) in *E. coli*. Specifically, we demonstrate that the SOS response acts as a crucial pathway to tightly control the expression of these IS*CR* transposases, *via* the LexA repressor.

## RESULTS AND DISCUSSION

### LexA binds to the putative promoter region of three IS*CR* transposase (*rcr*) genes

To investigate the expression of IS*CR* transposase genes (*rcr* genes), we first analyzed *in silico* the *ter*IS region sequences encompassing the promoter region of the *rcr* gene (Fig. 1A and B) of 17 nonredundant IS*CR* elements in *E. coli* (see Fig. S1). Interestingly, IS*CR1*, -*CR2,* and -*CR8* comprised a sequence matching the CTGT-N_8_-ACAG β-/γ-proteobacteria LexA-binding site consensus (LexA box) (19, 20), which overlaps with the −10 element of the predicted *rcr* transposase gene promoters (P*rcr*) (Fig. 1B and C), suggesting a potential regulation of these promoters by the repressor LexA. The LexA transcriptional factor is the master regulator of the SOS response, a global bacterial response to DNA damage (reviewed in reference 20), that acts as a repressor of SOS regulon genes under normal conditions. Upon DNA damages and the formation of single-strand DNA (ssDNA), the SOS response is triggered, through the formation of a nucleoprotein filament ssDNA-RecA that stimulates LexA autoproteolysis and, therefore, the expression of SOS-regulated genes.

**Fig 2:**
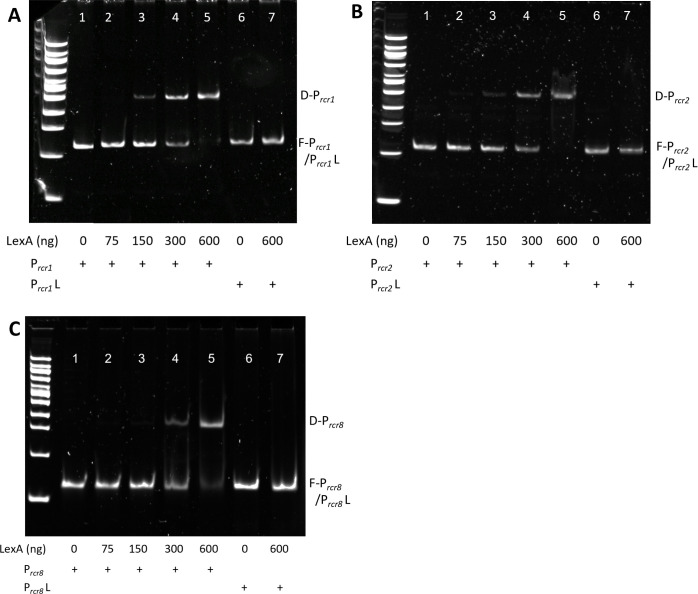
LexA binds to the *rcr* promoter region of IS*CR*1, IS*CR2,* and IS*CR8.* Electrophoretic mobility shift assays were performed with native *rcr* promoters (P*_rcr_* ; lanes 1–5) or LexA box-mutated *rcr* promoters (P*_rcr_* L; lanes 6–7) of *rcr1* (**A**), *rcr2* (**B**), or *rcr8* (**C**), in the presence or absence of purified LexA protein (amounts in nanograms are indicated). F, free DNA; D, delayed complex.

To confirm the functionality of the predicted LexA box in IS*CR1*, -*CR2,* and -*CR8*, we performed electrophoretic mobility shift assay (EMSA) of their *rcr* transposase promoter regions with purified *E. coli* LexA protein. We observed a DNA shift in the presence of the LexA protein for the three promoters P*_rcr_* (Fig. 2), showing that LexA specifically binds *in vitro* to the putative IS*CR* LexA box located in the native IS*CR1*, -*CR2,* and -*CR8* P*_rcr_* promoter regions. Conversely, no shift was detected when the putative IS*CR* LexA boxes were mutated in one of the conserved terminal triplet (CTGT-N_8_-ACAG into CTGT-N_8_-AACT; P*_rcr_*L) (Fig. 2). These results strongly suggest that LexA plays a role in regulating the expression of the corresponding *rcr* transposase gene and show that the sequences identified *in silico* are functional LexA boxes.

### The SOS response induces IS*CR1,* IS*CR2,* and IS*CR8* transposase expression

To characterize the SOS regulation of the *rcr* transposase genes *in vivo*, we constructed *lacZ* transcriptional fusions between the *lacZ* gene with its RBS and the *ter*IS regions of IS*CR1*, IS*CR2,* and IS*CR8*, including the putative *rcr* promoter (P*_rcr_*) sequence and LexA-binding site (Fig. 1D). We also constructed these fusions containing a mutation of the putative −10 box of the P*_rcr_* promoters (P*_rcr_* M) or the mutation in the LexA box (P*_rcr_* L) (Table 1).

**TABLE 1:** Bacterial strains and plasmids used in this study

Strain or plasmid	Genotype or description	Source or reference
Bacterial strains		
*Escherichia coli*		
MG1656	*lacZ-* derivative of *E. coli* K-12 MG1655	(21)
MG1656Δ*lexA*	Derivative of MG1656 with deletions of *sulA* and *lexA*	(22)
MG1656Δ*recA*	Derivative of MG1656 with deletion of *recA*	(23)
MG1656*lexA3*(Ind-)	Derivative of MG1656 carrying the *lexA3*(Ind-) allele encoding a non-cleavable LexA protein	Laboratory collection
BL21(DE3)/pLysS	F^–^ *ompT gal dcm lon hsdS _B_*(*r_B_*^–^*m_B_*^–^) (DE3), carrying the pLysS plasmid	(24)
BW25113/ICE*Pda*SpaI (ECI)	Strain carrying IS*CR2*	(25)
*Salmonella enterica* serovar Montevideo		
C1	Strain carrying IS*CR1*	Laboratory collection
*Pseudomonas* sp. ADP2		
PAD	Strain carrying IS*CR8*	(26)
Plasmids		
pSU38Δtot*lacZ*	Km^r^, vector carrying the promoterless *lacZ* gene	(27)
P*_rcr1_*	369-bp *ter*IS region of IS*CR1* and *lacZ* translation initiation signal cloned into pSU38Δtot*lacZ* (oligonucleotides CP69 and CP70 and C1 strain DNA)	This study
P*_rcr1_* M	P*_rcr1_* mutated in the −10 element of P*_rcr1_* with oligonucleotides CP69 and 4	This study
P*_rcr1_* L	P*_rcr1_* mutated in the LexA box of P*_rcr1_* with oligonucleotides CP69 and CP71	This study
P*_rcr2_*	106-bp *terI*S of IS*CR2* and *lacZ* translation initiation signal cloned into pSU38Δtot*lacZ* (oligonucleotides Prcr2L-EcoRI and CP72 and ECI strain DNA)	This study
P*_rcr2_* M	P*_rcr2_* mutated in the −10 element of P*_rcr2_* with oligonucleotides Prcr2-10mutL and Prcr2-10mutR	This study
P*_rcr2_* L	P*_rcr2_* mutated in the LexA box of P*_rcr2_* with oligonucleotides Prcr2L-EcoRI and CP73	This study
P*_rcr8_*	87-bp *ter*IS of IS*CR8* and *lacZ* translation initiation signal cloned into pSU38Δtot*lacZ* (oligonucleotides Prcr8L and Prcr8-lacZ and PAD strain DNA)	This study
P*_rcr8_* M	P*_rcr8_* mutated in the −10 element of P*_rcr8_* with oligonucleotides 15 and 16	This study
P*_rcr8_* L	P*_rcr8_* mutated in the LexA box of P*_rcr8_* with oligonucleotides Prcr8LexAmut2L and Prcr8LexAmut2R	This study
pUA1107p	pET15b with P_T7*lac*_::His_6_::*lexA_E.coli_-term*T7 pET expression system; P_T7*lac*_, N-terminal His_6_ tag, and Amp^r^	(23)
P*_sulA_*	*sulA* gene promoter region and *lacZ* translation initiation signal cloned into pSU38Δtot*lacZ*	(28)

**Fig 3:**
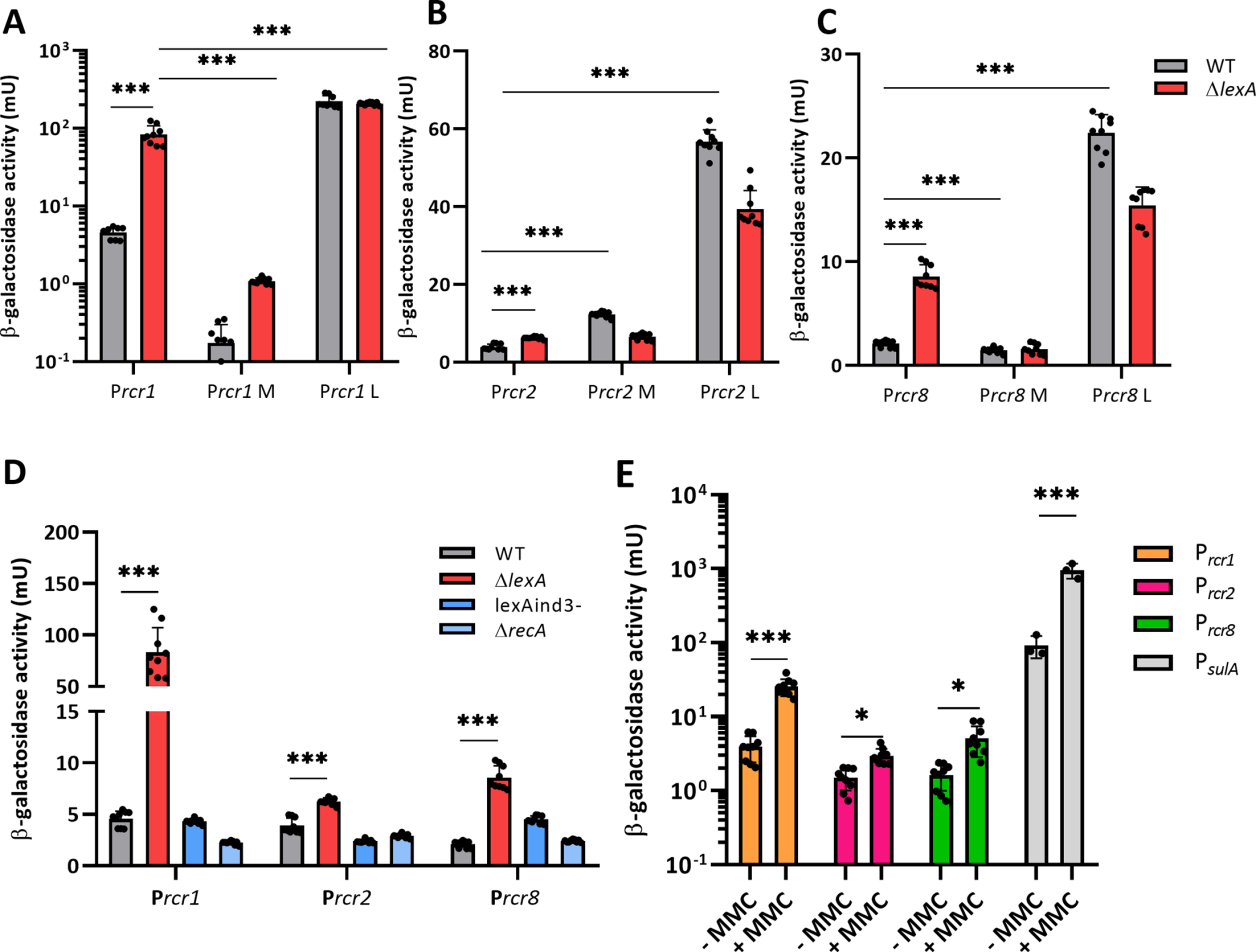
The SOS response controls the expression of IS*CR1*, IS*CR2,* and I*SCR8* transposase *rcr* genes. (**A–D**) LexA represses the expression of the transposase genes. β-galactosidase activities (in Miller units mU) were measured in the *E. coli* MG1656 reference strain (gray) and its Δ*lexA* (red), Δ*recA* (light blue), and *lexAind3-* (non-autoproteolytic LexA; turquoise) derivatives. These strains carry a plasmid containing a *lacZ* transcriptional fusion with the *rcr* promoter of IS*CR1* (**A, D**), IS*CR2* (**B, D**), or IS*CR8* (**C, D**) in either the native (P*_rcr_*), LexA-box-mutated (P*_rcr_*L), or −10 region-mutated (P*_rcr_* M) promoter configuration, as indicated. Experiments were performed three times in technical triplicates. Statistical significance was determined by unpaired two-tailed t tests (*** *P*-value < 0.01). (**E**) The IS*CR* transposase gene expression is induced by SOS response inducers. β-galactosidase activities were measured in the *E. coli* MG1656 reference strain carrying a plasmid containing a *lacZ* transcriptional fusion with P*_rcr1_* (orange), P*_rcr2_* (red), P*_rcr8_* (green), or P*_sulA_* (light gray; *E. coli SOS-*induced promoter, after treatment (+) or not (−) with mitomycin C (MMC), an SOS-inducing agent. All experiments were performed three times in technical triplicates. Statistical significance was determined by unpaired two-tailed t tests (*** *P*-value < 0.01, * *P*-value < 0.05). Graphs in panels A and E display a logarithmic y-axis.

The resulting recombinant plasmids were introduced into the *E. coli* K-12 MG1656 strain, its Δ*lexA* (constitutive expression of LexA-regulated promoters), and Δ*recA* (constitutive repression of LexA-regulated promoters) derivatives, as well as in a *lexA3*(Ind-) mutant encoding for an uncleavable LexA protein (29). Measuring β-galactosidase activities showed very low activity for all three promoters in the wild-type (WT) strain (<5 Miller units), suggesting that Rcr transposases are not constitutively expressed (Fig. 3A through C). These results are consistent with those in the literature. Indeed, weak promoter activity is a feature shared by many transposase gene promoters, including those from IS*21* (30), IS*30* (31), IS*911* (32), and IS*200* (33), to limit their impact on bacterial fitness. We observed a significant increase in β-galactosidase activity in the *lexA*-deleted strain, with varying strength for the three native promoters P*_rcr1_,* P*_rcr2,_* and P*_rcr8_* (18.2-, 1.6-, and 4.1-fold, respectively; Fig. 3A through C and Table S1). However, we did not observe a strong effect in conditions where the SOS response could not be induced (strains Δ*recA* and *lexA3*(Ind-)), with basal P*_rcr_* activities being very low (<5 Miller units) in the WT strain (Fig. 3D). Overall, the results are consistent with the potential regulation of P*_rcr1_*, P*_rcr2_*, and P*_rcr8_* by LexA. To further validate these results, we measured the β-galactosidase activities from P*_rcr_*L-mutated promoters (no binding of LexA). As expected, the mutation of the LexA-binding site led to a 48.5-, 14.5-, and 10.6- fold increase in β-galactosidase activities for P*_rcr1_*, P*_rcr2,_* and P*_rcr8_*, respectively (Fig. 3A through C and Table S1). These results demonstrate the involvement of LexA protein in the regulation of IS*CR1*, *CR2,* and *CR8 rcr* transposase gene promoters.

To confirm the SOS induction, we measured P*_rcr1_*, P*_rcr2,_* and P*_rcr8_* activity after treatment with mitomycin C (MMC), a DNA-damaging molecule known to induce the SOS response in *E. coli* (34, 35). As shown in Fig. 3E, treatment with MMC induced a significant and strong increase in β-galactosidase activity for P*_rcr1_* (7-fold) and milder ones for P*_rcr2_* and P*_rcr8_* (2- and 3.2-fold, respectively; Table S1).

The intrinsic activity of the three P*_rcr_* promoters was then analyzed to validate the σ^70^ putative promoter sequences identified *in silico* (Fig. 1B) by introducing mutations into the −10 region of the P*_rcr_* promoters (P*_rcr_* M ; see Materials and Methods). As anticipated from the very weak activity of the native promoters, P*_rcr_* M mutation almost completely abolished the activity of P*_rcr1_* and P*_rcr8_* in the Δ*lexA* background only, demonstrating the existence of an active promoter tightly repressed by LexA (Fig. 3A through C and Table S1). Surprisingly, for P*_rcr2_* mutated in the −10 region*,* we obtained a 3.1-fold higher β-galactosidase activity compared to P*_rcr2_*, in the wild-type strain. We did not observe such an increase in the Δ*lexA* strain (Fig. 3B). These results suggest either that the P*_rcr2_* promoter has not been correctly defined by the *in silico* approach (BPROM analysis [36]) or that the mutations performed to inactivate this promoter indeed disrupted its activity but allowed, at the same time, the recognition of a slightly more active promoter. We performed another *in silico* analysis confirming that introducing mutations in the initial putative −10 region of P*_rcr2_* allowed the identification of a new P*_rcr2_* promoter upstream of the initial one Fig. S2. This could explain the increased activity for P*_rcr2_* M in the wild-type background. This emphasizes two major facts: (i) introducing mutations to study regulation or expression can lead to a polarized effect; (ii) insertion sequence promoters can be plastic and display a range of regulation yet to be explored, such as recognition by alternative sigma factors, making them difficult to predict.

It should be noted that we searched only for σ^70^ promoter sequences to identify potential transposase *rcr* gene promoters *in silico*. The low levels of P*_rcr2_* activity observed under our experimental conditions could also reflect direct or indirect regulation of P*_rcr2_* by alternative sigma factors and/or other specific stress conditions beyond DNA damage, as reported for certain IS. A recent study showed that several ISs in *Deinococcus geothermalis* are induced and transpose during the exponential phase upon oxidative stress, depending on a noncharacterized alternative sigma factor (37). *Geobacillus kaustophilus* enhances IS transposition via an extracytoplasmic function sigma factor *sigX*-dependent stress responses at elevated temperatures when proliferative cells were prevented from active propagation (38). Interestingly, the Tn*4652* transposase gene promoter is controlled by the stationary-phase-specific sigma factor σ^S^ in *Pseudomonas putida* (39), and it is silent in *E. coli*, indicating that its expression requires a specific factor absent in *E. coli* (40).

### Implications for IS*CR* mobility

Although it is well known that the expression of genes involved in the mobility in many diverse MGEs, like prophages, plasmids, pathogenic islands, gene cassettes from integrons (reviewed in reference 41), and ICE (34), is controlled by the SOS response through LexA repression, only a few articles in the literature report the contribution of the host SOS system in the regulation of IS transposable elements. The SOS response has been involved in the UV-induced transposition of IS*10* (5) and possibly in the transposition of Tn*5* (IS*50*) in *E. coli* (42), but controversy persists regarding the role of RecA and LexA in regulating Tn*5* (43, 44). Here, we demonstrate that the expression of the transposases of IS*CR1* and IS*CR8* is directly and tightly regulated by the repressor of the SOS response, LexA. Although LexA can specifically bind to the *rcr* transposase promoter region of IS*CR2 in vitro*, P*_rcr2_* activity remains extremely low (below 5 Miller units) even when induced with SOS-inducing agents or in the Δ*lexA* mutant (less than 2-fold induction, Fig. 3B through E). By performing an *in silico* blastP search of the Rcr2 protein and analyzing the different Rcr2-encoding sequences, we found that 51% of the *rcr2* sequences were identified in *E. coli,* highlighting the relevance of using *E. coli* for studying the regulation of IS*CR2*. Furthermore, 84% of all the available *rcr2* sequences display a β-/γ-proteobacteria canonical LexA-binding sequence (Supplementary methods and Table S3). This strong conservation of the LexA site in the *rcr2* promoter region supports the existence of an SOS-dependent regulatory mechanism that is, however, likely associated with another regulatory mechanism, as discussed above. Intriguingly, IS*CR1* also appears to be restricted to β-/γ-proteobacteria (9), i.e., hosts mostly displaying a canonical SOS response.

Furthermore, the transposases encoded by IS*CR* elements are members of the HUH family of endonucleases that use as a substrate an ssDNA known to trigger the SOS response. Thereby, the availability of the ssDNA substrates for IS*CR* transposase may be sensed as a signal to stimulate the transposase expression through the induction of the SOS response, ultimately promoting their transposition.

Although the transposition mechanism of the IS*CR* elements has not been experimentally proven, the present study demonstrates that under DNA-damaging conditions, the transposase genes of IS*CR1*, -*CR2,* and -*CR8* elements can be expressed. While their level of expression remains low, even when derepressed, they could contribute to the spread of IS*CR* and adjacent AR genes. A recent study indeed highlighted that the very low promoter activity of the transposase gene of IS*1* (around 15 Miller units) from a single copy on the chromosome can lead to high levels of its *cis*-transposition. The authors also show that high levels of transposase allow *trans*-activity on other normally nonmobile IS*1* elements and that the *trans*-transposition frequency is proportional to the levels of transposase expressed (45).

In a previous study, we analyzed *in silico* the diversity of IS*CR1 ori*S downstream genes and found that 84% of the analyzed sequences (946 out of 1,127) carried a gene downstream of the transposase gene *rcr1*, encoding for known or putative ARGs conferring resistance to five antibiotic families: trimethoprim (*dfrA19*, *dfrA3*, etc.), β-lactams (*bla*_CTX-M-9_, *bla*_CMY-9_, *bla*_PER-1_, etc.), quinolones (*qnrA1*, *qnrA3*, and *qnrVC6*), chloramphenicol (*catA2*, putative *catA,* and *floR*), and aminoglycosides (*aphA6* and *rmtB*) (9). Here, we analyzed *in silico* 325 sequences of IS*CR2* (Table S3). Contrary to the diversity of genes found downstream of IS*CR1*, several identical combinations of genes are present upstream and downstream of IS*CR2*, with four combinations being over-represented, containing *floR* or *sul2* resistance genes. i) *floR*, DUF3363, IS*CR2*, putative/LysR-like, and recombinase/resolvase; ii) *floR*, LysR-like, IS*CR2*, putative, and *sul2*; iii) *sul2*, *glmM*, IS*CR2*, and putative; and iv) *sul2*, putative, IS*CR2*, and putative/LysR-like (Table S3). These two genes confer resistance to florfenicol and sulfonamides, respectively. While the *floR* gene is primarily found in animals (46), *sul2* is found in both clinical settings (47) and in animals (48). For IS*CR8*, we did not find any ARG downstream of the *rcr8* gene, but the small number of full sequences of the *rcr8* transposase gene in the database (*n* = 9, Table S3) does not allow us to draw any conclusions on its ability to mobilize DNA fragments.

The *rcr2* gene has been frequently associated with the SXT ICE (8), and our *in silico* analysis also showed that IS*CR2* is often found on a plasmid or combined with a recombinase/resolvase gene (Table S3). Given the extremely low level of expression of IS*CR1, -CR2,* and -*CR8 rcr* transposases, even when derepressed, these IS*CR* elements may also rely on the MGE carrying them, such as plasmids or ICE (for IS*CR2*), to spread.

This study demonstrates, in *E. coli*, a direct regulation of the expression of the transposase gene by the SOS response and its repressor LexA, in three members of the IS*CR*/IS*91* family of bacterial IS elements. As we witness an ever-increasing spread of antibiotic resistance globally, understanding the mechanisms that govern the emergence of ARGs is critical. In this context, our findings reveal the importance of the host SOS response in regulating IS*CR* expression and offer evidence that, by relying on host-regulatory mechanisms, these elements exhibit a β-/γ-proteobacteria host specificity. Such SOS-dependent repression limits the production of a likely costly transposase, while ensuring that the transposase gene required for mobility is expressed only under specific conditions, and potentially allows the host bacteria to adapt to antibiotic pressure.

A further study will be conducted to determine whether IS*CR1* mobility increases in response to stress caused by DNA damage.

## MATERIALS AND METHODS

### Bioinformatic analysis of IS*CR* elements

Seventeen IS*CR* members considered in this study were recovered from the initial list published by Toleman et al., completed by the current updated list from the Galileo database, and curated to discard IS*CR* elements sharing more than 98% nucleotide identity (8, 49). When available, we aligned the sequences upstream of the *rcr* transposase START codon up to 150 bp. Putative σ^70^ promoters were predicted by BPROM (36). Alignments were performed using Clustal Omega (https://www.ebi.ac.uk/jdispatcher/msa/clustalo) (50) with default parameters. Sequences and accession numbers of all IS*CR* used for this alignment are given in Fig. S1.

### Bacterial strains and plasmids

Bacterial strains and plasmids are listed in Table 1. Bacterial strains were grown in lysogeny broth (LB) at 37°C. The medium was supplemented with kanamycin (25 µg/mL) when required. The expression of *E. coli* His_6_::LexA protein in *E. coli* BL21(DE3)/pLysS was induced by adding 1 mM isopropyl-β-D-thiogalactopyranoside (IPTG) to the medium.

### DNA manipulation

Plasmid DNA was extracted from *E. coli* using the Wizard Plus SV Minipreps DNA Purification Kit (Promega, Madison, WI, United States), and genomic DNA was extracted using the SaMag Bacterial DNA Extraction Kit (Sacace, Biotechnologies). PCRs were carried out with Phusion DNA Polymerase (Thermo Fisher Scientific) to amplify fragments subsequently used for cloning or with the Taq’Ozyme Purple Mix 2 (Ozyme) to screen transformants. Oligonucleotides used in this study are listed in Table S2. PCR products were loaded and visualized on 1.6% agarose gel, extracted, and purified with the Wizard ^SV^ Gel (Promega, Madison, WI, United States) or PCR Clean-Up kit (Macherey-Nagel).

### Plasmid constructions

Transcriptional *lacZ* fusions were constructed in the pSU38Δtot*lacZ* reporter plasmid (27). The *rcr* promoter region amplified from bacterial strains known to carry the corresponding IS*CR* was cloned into the *EcoR*I/*BamH*I unique restriction sites of pSU38Δtot*lacZ* (see Table 1). Mutations in LexA boxes were performed by PCR assembly to convert the LexA box essential triplet “CAG” into “ACT,” as previously done (22). The P*_rcr_* promoters were mutated by PCR assembly in their putative −10 elements: from TACTGT to **CG**CTGT (IS*CR1*; P*_rcr1_* M), TAGACT to **CG**GACT (IS*CR2*; P*_rcr2_* M), and TACTGT to **GC**CTGT (IS*CR8*; P*_rcr8_* M).

### β-Galactosidase assays

β-Galactosidase assays were performed in the *E. coli* MG1656 strain from at least three independent assays and three technical replicates for each construct, as previously described, at 37°C (27). The SOS response was induced with the addition of mitomycin C (MMC) 1.6 µg/mL in the culture 1 hour before reaching 0.6–0.8 OD_600_ (22).

### Electrophoretic mobility shift assay (EMSA)

Over-expression and purification of the *E. coli* His_6_::LexA protein was performed as described previously (51). EMSA probes, with natural (P*_rcr_*) or mutated LexA-binding site (P*_rcr_* L), were amplified by PCR from the C1 (IS*CR1*) or ECI (IS*CR2*) bacterial strains (Table 1) using oligonucleotide pairs ISCR1-GS-L/ISCR1-GS-R, ISCR1-lexAmutL/ISCR1-lexAmutL, Prcr2L-EcoRI/ISCR2_GS_R, and Prcr2-LexAmutL/Prcr2-LexAmutR for probes P*_rcr1_,* P*_rcr1_* L (218 bp), P*_rcr2,_* and P*_rcr1_* L (209 bp), respectively (Table S2). EMSA probes were purified on agarose gel 1.6%. For P*_rcr8_*, the WT and mutated probes were ordered from Merck as bottom and top strands (C129+CP130 and CP131+CP132, respectively) (Table S2) that were assembled prior to the EMSA experiment.

Increasing quantities of recombinant His_6_::LexA protein (0 to 600 ng) were incubated on ice for 20 min with 40 ng of DNA probes in a final volume of 10 µL of binding buffer (10 mM HEPES pH 8, 10 mM Tris-HCl pH 8, 50 mM KCl, and 1 mM EDTA). Then, 2 µL of 6X EMSA gel-loading solution (EMSA Kit, Molecular Probes BioRad) was added to the reaction mixture. Samples were separated in 6% nondenaturing Tris-glycine polyacrylamide gels and visualized with SYBR Green following the manufacturer’s protocol (EMSA Kit, Molecular Probes BioRad).

### Statistical analysis

Statistical analysis was performed using the *t*-test with two unpaired groups.

## SUPPLEMENTAL MATERIAL

Table S1 jb.00551-25-s0001.pdf Raw data for graphs in article figures.

Supplemental Methods; Table S2; and Figures S1 to S2 jb.00551-25-s0002.docx Supplemental methods, *in silico* study of complete *rcr2* and *rcr8* genes and their genetic environment; Table S2, oligonucleotides; Fig. S1, sequences used for alignment; Fig. S2, mutations in promoter sequences.

Table S3 jb.00551-25-s0003.xlsx *In silico* study of ISCR2 and ISCR8 genetic environment.

ASM does not own the copyrights to Supplemental Material that may be linked to, or accessed through, an article. The authors have granted ASM a non-exclusive, world-wide license to publish the Supplemental Material files. Please contact the corresponding author directly for reuse.
