# Supplementary material for: The bacterial SOS response promotes the expression of the transposase encoded by ISCR mobile genetic elements
Source: J Bacteriol. 2026 Jun 23;208(7):e00551-25. doi: 10.1128/jb.00551-25 (PMC13393415; doi:10.1128/jb.00551-25)
Supplement: Table S1 — Raw data for graphs in article figures. [file jb.00551-25-s0001.pdf]

ISCR1 (Fig.3A)

beta-galactosidase activity (miller units)

	**WT**									**Δ*lexA***								
**P*rcr1***	4,98	5,17	5,46	3,62	3,62	3,57	4,66	4,77	5,19	91,43	124,9	116,2	78,31	82,22	75	58,05	64,44	58,46
**P*rcr1* M**	0	0	0,1	0,19	0,19	0,33	0,35	0,23	0,18	1,15	1,04	1,27	0,98	1,02	1,06	0,97	1,16	1,14
**P*rcr1* L**	187,68	184,9	195,71	252,4	280,3	284,4	203,6	202,9	199,7	215,7	217,9	216,9	210,8	213,3	206	192,9	195,8	196,6

	WT		ΔlexA		ratio compared to prcr1		ratio LexA/ WT
	average	SD	average	SD	WT	ΔlexA	
P*rcr1*	4,56	0,75	83,23	23,95			18,25
P*rcr1* M	0,17	0,13	1,09	0,10	0,04	0,01	6,24
P*rcr1* L	221,30	39,78	207,31	9,86	48,53		0,94

ISCR2 (Fig.3B)

beta-galactosidase activity (miller units)

	**WT**									**Δ*lexA***								
**P*rcr2***	3,23	3,44	3,28	3,28	3,49	3,79	4,9	4,96	4,75	6,62	6,53	6,33	5,65	5,97	6,21	6,22	6,14	6,71
**P*rcr2* M**	11,57	12,31	12,54	13,14	11,95	10,84	13,01	12,78	12,36	6,77	6,91	7,16	7,21	7,09	7,57	5,66	5,54	5,39
**P*rcr2* L**	55,14	55,83	51,18	62,16	59,33	57,87	56,93	55,44	56,51	40,78	49,36	44,82	36,39	35,77	35,5	37,37	36,77	37,56

	WT		ΔlexA		ratio compared to Prcr2		ratio LexA/ WT
	average	SD	average	SD	WT	ΔlexA	
P*rcr2*	3,90	0,75	6,26	0,33			1,61
P*rcr2* M	12,28	0,73	6,59	0,83	3,15	1,052	0,54
P*rcr2* L	56,71	3,03	39,37	4,77	14,53		0,69

ISCR8 (Fig.3C)

beta-galactosidase activity (miller units)

	WT									Δ*lexA*								
**P*rcr8***	1,66	1,68	1,65	2,29	2,46	2,36	2,32	2,31	2,22	7,77	7,72	7,61	8,7	7,96	7,4	10,26	10,04	9,69
**P*rcr8* M**	0,99	1,22	1,28	1,55	1,92	1,62	1,66	1,45	1,63	1,61	1,44	1,57	1,06	1,05	0,96	2,04	2,2	2,28
**P*rcr8* L**	20,99	24,5	22,59	24,05	23,78	23,39	20,51	22,61	19,36	13,37	12,67	13,28	16,72	16,18	16,07	16,94	16,88	16,81

	WT		ΔlexA		ratio compared to Prcr8		ratio LexA/WT
	average	SD	average	SD	WT	ΔlexA	
P*rcr8*	2,11	0,34	8,57	1,14			4,07
P*rcr8* M	1,48	0,28	1,58	0,51	0,70	0,18	1,07
P*rcr8* L	22,42	1,76	15,44	1,78	10,65		0,69

recA lexAi3nd (Fig.3D)

beta-galactosidase activity (miller units)

	**WT**									**ΔlexA**								
**P*rcr1***	4,98	5,17	5,46	3,62	3,62	3,57	4,66	4,77	5,19	91,4	125	116	78,3	82,2	75	58,1	64,4	58,5
**P*rcr2***	3,23	3,44	3,28	3,28	3,49	3,79	4,9	4,96	4,75	6,62	6,53	6,33	5,65	5,97	6,21	6,22	6,14	6,71
**P*rcr8***	1,66	1,68	1,65	2,29	2,46	2,36	2,32	2,31	2,22	7,77	7,72	7,61	8,7	7,96	7,4	10,3	10	9,69

	**lexAind3-**									**Δ*recA***								
**P*rcr1***	4,43	4,78	4,46	3,91	4,41	4,35	4,13	4,13	4,27	2,32	2,29	2	2,28	1,9	2,32	2,48	2,23	2,38
**P*rcr2***	2,47	2,47	2,74	2,35	2,32	2,35	2,22	2,28	2,23	3,26	2,87	2,93	2,72	2,61	2,75	3,03	3,05	2,98
**P*rcr8***	4,4	4,52	4,27	4,37	4,07	4,02	4,85	4,96	4,94	2,62	2,5	2,31	2,55	2,3	2,26	2,47	2,44	2,3

	WT		ΔlexA		lexAind3-		ΔrecA	
	average	SD	average	SD	average	SD	average	SD
P*rcr1*	4,56	0,75	83,23	23,95	4,32	0,25	2,24	0,18
P*rcr2*	3,90	0,75	6,26	0,33	2,38	0,16	2,91	0,20
P*rcr8*	2,11	0,34	8,57	1,14	4,49	0,36	2,42	0,13

Induction MCC (Fig.3E)

beta-galactosidase activity (miller units)

	P_{rcr1}									P_{rcr2}								
**- MMC**	3,84	3,94	3,93	2,26	2,49	2,06	6,16	6,11	4,43	1,99	2,02	2,03	0,73	1,18	0,90	1,69	1,53	1,39
**+ MMC**	39,10	20,20	17,23	26,54	28,42	30,26	25,12	21,47	21,66	4,44	2,59	3,04	3,08	2,78	3,69	2,34	2,30	2,29

	P_{rcr8}									P_{sfiA}		
**- MMC**	0,97	0,85	0,72	2,35	2,08	2,38	1,80	1,66	1,84	77,10	127,40	71,82
**+ MMC**	3,39	3,14	2,41	8,67	8,64	6,30	5,15	4,31	4,14	1166,37	959,69	730,11
