# Supplementary material for: The bacterial SOS response promotes the expression of the transposase encoded by ISCR mobile genetic elements
Source: J Bacteriol. 2026 Jun 23;208(7):e00551-25. doi: 10.1128/jb.00551-25 (PMC13393415; doi:10.1128/jb.00551-25)
Supplement: Supplemental Methods; Table S2; and Figures S1 to S2 — Supplemental methods, in silico study of complete rcr2 and rcr8 genes and their genetic environment; Table S2, oligonucleotides; Fig. S1, sequences used for alignment; Fig. S2, mutations in promoter sequences. [file jb.00551-25-s0002.docx]

**Supplementary data to**

**The bacterial SOS response promotes the expression of the transposase encoded by IS*CR* mobile genetic elements**

Claire Lallement^1^, Thomas Jové^1^, Cécile Pasternak^1+^, Sandra Da Re^1*^ and Marie-Cécile Ploy^1*^

^1^**Univ. Limoges, INSERM, CHU Limoges, RESINFIT, U1092, F-87000 Limoges, France**

*To whom correspondence should be addressed. Tel: +33 5 19 56 42 63

Email: [marie-cecile.ploy@unilim.fr](mailto:thomas.jove@unilim.fr); [sandra.da-re@unilim.fr](mailto:sandra.da-re@unilim.fr)

**Supplementary Methods**

*In silico* study of complete *rcr2* and *rcr8* genes and their genetic environment.

Each protein of *rcr2* and *rcr8* had been blasted on blastP (ClusterN) at NCBI website and hits with 100% coverage and down to 98% of identity had been retrieved (01/27/2026 for IS*CR2* and 02/06/2026 for IS*CR8*). For both IS*CR2* and IS*CR8* direct genetic environment had been identified: 2 genes upstream (US_1, US_2) and 2 genes downstream (DS_1, DS_2). Source (animal, human, environment or unknown) from where DNA had been isolated from as well as bacterial species had been gathered too.

For the analysis of the SOS boxes: When promoter regions were available, we searched for the LexA-box sequence: if present, the sequence was added to the “SOS box” column in Table S3; if absent, we indicated “none”. The absence of the promoter region in the retrieved sequences, is indicated as a “-“ sign in the “SOS box” column of Table S3. The percentage of IS*CR2* carrying a LexA box was calculated based on sequences containing the promoter region i.e. 286 of the 325 selected sequences. All data were compiled in Table S3.

**Table S2:** Oligonucleotides used in this study

| **Name** | **Sequence (5’→ 3’)** |
| --- | --- |
| CP69 | CCGGAATTCATATCTCCTTTTGGGTTG |
| CP70 | GCGGGATCCATGGCTGTTTCCTGCTGATACTGTGGTTATGTAC |
| Prcr1-mutL | TGGACGTCTGAACGCAAGCCGCTGACGCTGTACATAACCACAGT |
| Prcr1-10mutR | GATACTGTGGTTATGTACAGCGTCAGCGGCTTGCGTTCAGACG |
| ISCR1-lexAmutL | CGCTGATACTGTACATAACCAACTTATCAGCAGGAAACAG |
| CP71 | GCGGGATCCATGGCTGTTTCCTGCTGATAAGTTGGTTATGTAC |
| Prcr2L-EcoRI | CCGGAATTCGGGAGTGACGGGCACTGGC |
| CP72 | GCGGGATCCATGGCTGTTTCCTGCAATACTGTGTTTACATAC |
| Prcr2-10mutL | AAAAGAACTTTCCGCTAAGCGACGGACTGTATGTAAACACAGTATTG |
| Prcr2-10mutR | TCCTGCAATACTGTGTTTACATACAGTCCGTCGCTTAGCGGAAAGT |
| Prcr2-LexAmutL | TAAGCGATAGACTGTATGTAAACAACTTATTGCAGGAAACAG |
| CP73 | GCGGGATCCATGGCTGTTTCCTGCAATAAGTTGTTTACATAC |
| Prcr8L | CCGGAATTCACAAGGCCCGAACTGGCGAC |
| Prcr8-lacZ | GCGGGATCCATGGCTGTTTCCTGAATGCTGTACAAATG |
| Prcr8mut-10L | CAAACAATCGCCGCAGAAGCCTGTGCATTTGTACAGCA |
| Prcr8-10mutR | CTGTACAAATGCACAGGCTTCTGCGGCGATTGTTTGGG |
| Prcr8LexAmut2L | AACAATCGCCGCAGAATACTGTGCATTTGTAACTCATTCA |
| Prcr8LexAmut2R | CGCGGATCCATGGCTGTTTCCTGAATGAGTTACAAATGCACAG |
| CP47 | ATTAGGATCCATATCTCCTTTTGGGTTG |
| CP50 | TCATCCTGCAGGGTTCGGGTATAGGAAGTATAAAC |
| CP49 | ATTAGGATCCTCGCTCAACCGGTAGTGGC |
| CP51 | TTACCCTGCAGGTCGTCAGTCAAAGAGACGACTC |
| CP55 | CCGCCTGCAGGTCAGTCAAAGAGACGACTC |
| ISCR1-GS-L | CAACCGGTAGTGGCTGAT |
| ISCR1-GS-R | CAGCGTCTGGTCGGGTTG |
| ISCR2_GS_R | AAGGGTTTGCTCGGGTCG |
| pZA2UP | TCCCAACCTTACCAGAGGGC |
| Fwd-lacZ | CGCCAGGGTTTTCCCAGTCAC |
| MRVD2 | TTCTGCTGACGCACCGGTG |
| PUP-ISCR2L | CCGGAATTCACCTTGAGACAGAACGCG |
| PqacED1L-EcoRI | CGCGAATTCTTAGATGCACTAAGCACATAATTGCTCACAG |
| Prcr2-LexAmutR | GCGTCCTTGCAATAAGTTGTTTACATACAGTCTATCGCT |
| CP129 | GGCGACGGTTCGGGCCTTGTGCTTTGCGTACCCAAACAATCGCCGCAGAATACTGTGCATTTGTACAGCATTCATGTCAGCCACTTCCAAGCCCAAGCTCTACAACCCACGCCACCCCGA |
| CP130 | TCGGGGTGGCGTGGGTTGTAGAGCTTGGGCTTGGAAGTGGCTGACATGAATGCTGTACAAATGCACAGTATTCTGCGGCGATTGTTTGGGTACGCAAAGCACAAGGCCCGAACCGTCGCC |
| CP131 | GGCGACGGTTCGGGCCTTGTGCTTTGCGTACCCAAACAATCGCCGCAGAATACTGTGCATTTGTAACTCATTCATGTCAGCCACTTCCAAGCCCAAGCTCTACAACCCACGCCACCCCGA |
| CP132 | TCGGGGTGGCGTGGGTTGTAGAGCTTGGGCTTGGAAGTGGCTGACATGAATGAGTTACAAATGCACAGTATTCTGCGGCGATTGTTTGGGTACGCAAAGCACAAGGCCCGAACCGTCGCC |

**Figure S1: Sequences and accession number of IS*CR* family members used for Figure 1B alignment.**

>IS*CR1* **AM234698**

AAACCTTCTCGCTCAACCGGTAGTGGCTGATAACAACTCGTGAGGGCTATTGCGGGTTAAGCATTTAGCGATGTCTAGGGCCAGACTGGACGTCTGAACGCAAGCCGCTGATACTGTACATAACCACAGTATCAGCGGAGGATACCCATG

>IS*CR2* **AY055428**

TTTAGCCCAGAGGAACGCCCAGACGACAGGGAGTGACGGGCACTGGCTGGCAATGTCTAGCAACGGCAGGCATTTCGGCTGAGGGTAAAAGAACTTTCCGCTAAGCGATAGACTGTATGTAAACACAGTATTGCAAGGACGCGGAACATG

>IS*CR3* **KT935446**

AGGACGGGGCCGGAGCCCGGCAGCGATGCCGGGCTTTTTGTTGTGCCCGCGCCGCGGCAATGTCTGACGCGAAGATCAGAACGCACCGATACGAACGTGCGAACACAGGCGCAACACTGAGCAGCCGTCCCCGCACCGGAGCGCTGCGTG

>IS*CR4* **AY341249**

ATTGCCTGCCCGCGGCGCGGCGATTTCCGAGCCGCGTATCAGGGAGACGCCGATGTGACGGCGTGACCGTGGGCGCAACCCTGTGCTGCCGTCGCTGCACAGGAGCGTTCCGTGCCGCAGCTCATCGCACTCCGGCGCGAGCCCGTCGTG

>IS*CR5* **AM849110**

CTCTGGGCCGCGCCCTGCACCTGGGTGTCGAGGCGGTCGAGGCGGGAGTCGCCGCGGCAATGTCTGACGCGAAGATCAGAACGCACCGATACGAACGTGCGAACACAGGCGCAACACTGAGCAGCCGTCCCCGCACCGGAGCGCTGCGTG

>IS*CR6* **GQ388247**

AGGACGGGGCCGGAGCCCGGCAGCGATGCCGGGCTTTTTGTTGTGCCCGCGCCGCGGCAATGTCTGACGCGAAGATCAGAACGCACCGATACGAACGTGCGAACACAGGCGCAACACTGAGCAGCCGTCCCCGCACCGGAGCGCTGCGTG

>IS*CR7* **AJ250371**

CGTCGAGCGCAGCTTCCGCCGCTATCTCGAATGCGGCATCCTCGCCCACGGCTTTGCCCGCGCCTGGTGTGACACCTGCCAGCATGAGTTCCTCATCGCCTATTCCTGTAAAGGGCGGGGCGTATGCCGTCCTGCAACACGCGGCGCATG

>IS*CR8* **AF028594**

GCTGAAGATCACCGAGGCGGTCGTCCTGCGCGCAAGTAAGTCCGCAGCCAAGCTACCCTGACAAGGCCCGAACTGGCGACGGTTCGGGCCTTGTGCTTTGCGTACCCAAACAATCGCCGCAGAATACTGTGCATTTGTACAGTATTCATG

>IS*CR10* **AXRA01000008**

TGCCGGGCTTTGTGCGTTCTGGCGTCCGAGGCGGGAGACTTCCTACCCGCCCCGCGGCAATGTCTGACGCGAAGATCAGAAAACGCCGATATGAACGCGTGCTCGCGGGCGCAACCCTGAGCAGCCGTCCCTGCAACGGAGCGCTGCGTG

>IS*CR14* **LC549808**

AGGACGGGGCCGGAGCCCGGCAGCGATGCCGGGCTTTTTGTTGTGCCCGCGCCGCGGCAATGTCTGACGCGAAGATCAGAACGCACCGATACGAACGTGCGAACACAGGTGCAACCCTGAGCAGCCGTCCCCGCACCGGAGCGCTGCGTG

>IS*CR15* **AP017302**

GAGCGGACGTAAAGCCCCTCCTCCCCGCCCGCACCCTCGGCGGCCGCGGCGTGGGCATGGAAAAGCACTACACCGACTGGTTCGAGCGCATCCTCCCCGACACCCTCACCATCCGCGATGTGCGAGCACTGCCGTGCAGAGAGGCTGGTG

>IS*CR16* **CP175593**

TGCCGGCCGGCGGCGGCATGGGCGGCATGGGCGGCATGGATTTCTAAGCCCCGCGATCCATCAAGCAAGACCACAAAGCCCGGCCTCGTGCCGGGCTTTGTGCGTTCTGGCGTCCGAGGCGGGAGACTTCCTACCCGCCCCGCGGCAATG

>IS*CR19* **EU503121**

TGCCGGGCTTTGTGCGTTCTGGCGTCCGAGGCGGGAGATTTCCTACCCGCCCCGCGGCAATGTCTGACGCGAAGATCAGAAAACGCCGATATGAACGCGTGCTCGCGGGCGCAACCCTGAGCAGCCGTCCCTGCAACGGAGCGCTGCGTG

>IS*CR20* **GU441460**

ACGCCCTGTGCGGACCCGCACGCAGGGTGTTGTGGGGGCTGGGGGCTAGAAACCCCCGGCTACCCGATTAGGTGGCGTCAGTCCACATTTTCCGCTTTTTTCTTCCAAATTGCCAGTAAATGTTCGTTTTTCTGCGTATGTATATATATG

>IS*CR21* **CP003022**

AGGCGTCAACCTAACTTTCGGTTGAGCTGCGAACGGGCTTGTCCCGTGAGTCCGCTCGAACCGGTTGTTAGGTGGCGCCAGTCCACATTTTCCATTATTTTCTTCGCAATTGCAAGCAAATGATCGTTTTCCTGCGTATGTATAGGTATG

>IS*CR22* **AY831462**

CTGATGTTCGTTAAAGCGGTTTCATAAAACGGTGGTTTTATCGTACTCCCCAGACAGAGCGCTGGTTTGCGGGCAGGTACTGGGCTTAAGTGCGTTTACTGTTCCATTGCTCCCCGAACCCCAATACTGTGTATTTGCACAGTATTCATG

>IS*CR24* **HQ541434**

TGTTGTGCCCGCGCCGCGGCAATGTCTGACGCGAAGATCAGAACGCACCGATACGAACGTGCGAACACAGGCGCAACACTGAGCAGCCGTCCCCGCACCGGAGCGCTGCGTGCCGCGCCTCGCCACATCCCGGCGGCAAGCCGCGGGATG


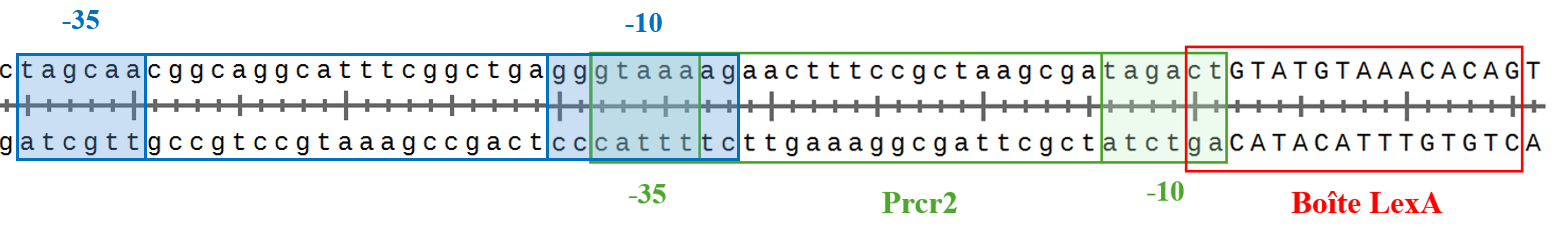
**Figure S2: Mutation in -10 region generates an alternative promoter in P*_rcr2_***

**LexA Box**

Introduction of mutation within the -10 region of the potential P_r_*_cr2_* (green) can generate a new alternative promoter highlighted in blue as identified by Softberry software (BPROM ; http://www.softberry.com/berry.phtml?topic=bprom&group=programs&subgroup=gfindb).
